# Carotid CTA with automated plaque quantification for risk stratification of coexisting obstructive coronary artery disease

**DOI:** 10.1186/s13244-026-02472-5

**Published:** 2026-11-15

**Authors:** Lusiying Xu, Minjie Zhao, Changsheng Zhou, Jianan Li, Sijia Zhao, Jianrui Li, Hao Tang, Sui Chen, Rui Liu, Wusheng Zhu, Ya Liu, Yun Zhang, Qiji Jin, Wenjing Cui, Pengbo Jiang, Dijia Wu, Xiaoqing Cheng, Longjiang Zhang

**Affiliations:** 1 School of Medical Imaging, Xuzhou Medical University, Xuzhou, China; 2 Department of Medical Imaging, Nanjing Jinling Hospital, Affiliated Hospital of Medical School, Nanjing University, Nanjing, China; 3 Department of Neurology, Nanjing Jinling Hospital, Affiliated Hospital of Medical School, Nanjing University, Nanjing, China; 4 Department of Research and Development, Shanghai United Imaging Intelligence Co. Ltd., Shanghai, China

**Keywords:** Carotid stenosis, Plaque, Automation, Computed tomography angiography, Coronary artery disease

## Abstract

**Objectives:**

To evaluate whether automated carotid plaque quantification on carotid computed tomography angiography (CTA) can serve as an adjunctive risk stratification tool for identifying patients with coexisting obstructive coronary artery disease (OCAD).

**Materials and methods:**

This dual-center retrospective study included 2533 adults undergoing paired carotid and coronary CTA within 3 months. Carotid plaque components (calcified, fibrotic, and lipid) were quantified automatically. In the modeling cohort, three diagnostic models for OCAD (stenosis ≥ 50%) were developed using LASSO regression: clinical, plaque, and integrated models. Performance was assessed by AUC (with DeLong’s test), decision curve analysis, and calibration curves, with validation in an internal validation set and a temporally independent validation cohort. Sensitivity analysis was performed using severe stenosis (≥ 70%) as the outcome.

**Results:**

All carotid plaque volumes were significantly associated with OCAD (*p* < 0.001). Calcified plaque volume (OR, 2.43; 95% CI: 1.95–3.04) and fibrotic plaque volume (OR, 1.37; 95% CI: 1.01–1.85) were independent predictors. The plaque model achieved an AUC of 0.785 (95% CI: 0.761–0.810) in the training set, comparable to the integrated model (AUC, 0.800) and superior to the clinical model (AUC, 0.723; *p* < 0.001). Performance remained robust in the internal (AUC, 0.824) and external validation set (AUC, 0.813). The integrated model demonstrated good calibration. Sensitivity analysis yielded consistent results.

**Conclusion:**

In this retrospective study, automated carotid plaque quantification on CTA provides an adjunctive risk stratification tool to identify patients at high risk for coexisting OCAD, potentially guiding decisions regarding downstream coronary examinations.

**Key Points:**

***Question*** Quantitative analysis of carotid plaque components on CTA may help identify patients with OCAD.

***Findings*** A model integrating automated carotid plaque volumes and clinical factors effectively identified OCAD.

***Clinical Relevance*** This study demonstrates that automated carotid plaque quantification on CTA may serve as an adjunctive risk stratification tool to guide downstream coronary testing in patients with cerebrovascular disease.

**Graphical Abstract:**

**Figure:**
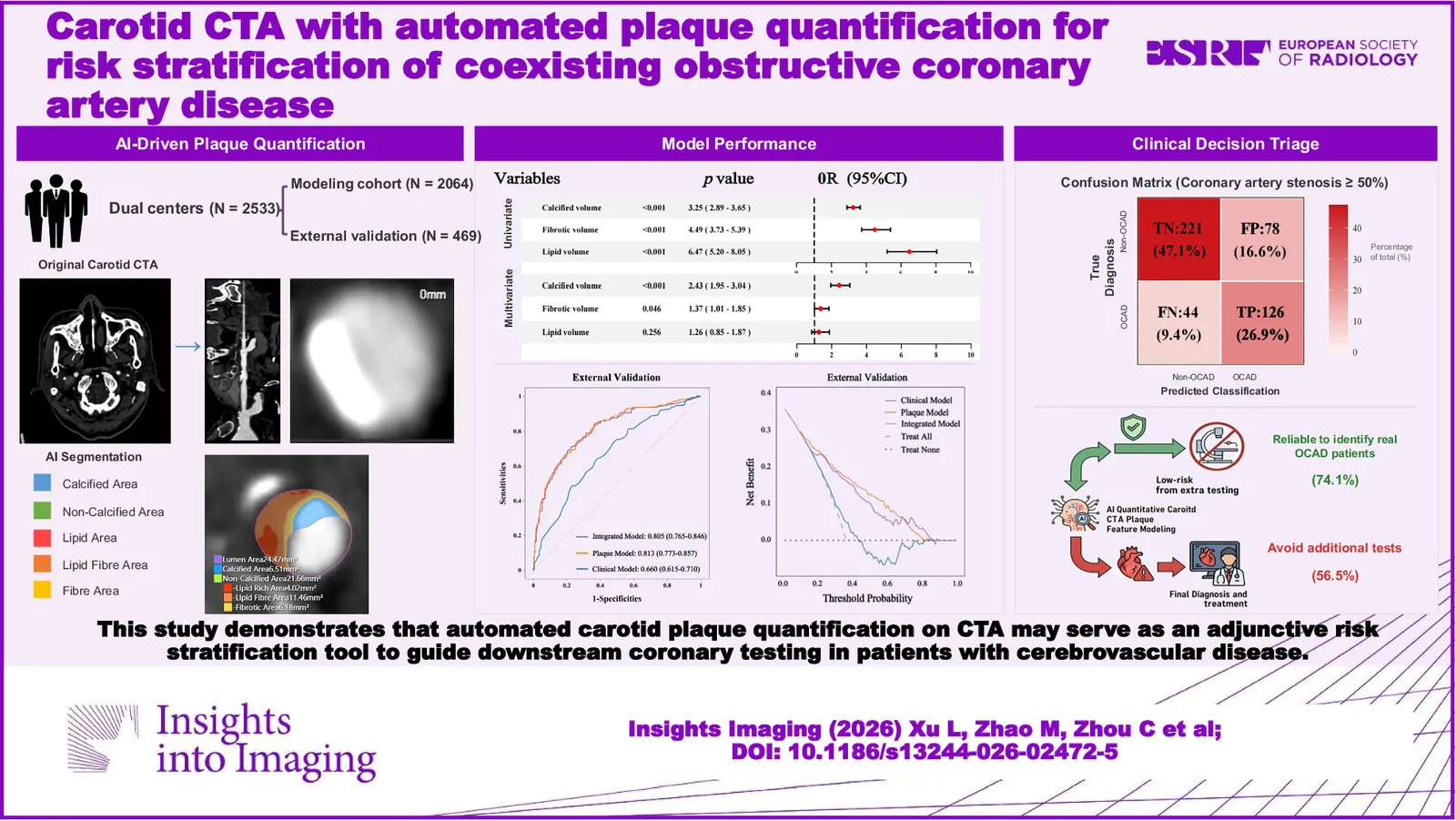

## Introduction

Atherosclerosis is a systemic vascular disorder that commonly underlies the coexistence of cerebrovascular and cardiovascular disease [1]. Approximately one-third of patients with severe carotid artery stenosis harbor concomitant coronary artery disease (CAD) [2], and this proportion rises to 63.4% among those undergoing carotid revascularization [3]. Such cardio-cerebrovascular comorbidity substantially elevates the risk of perioperative cardiac events during invasive procedures, including carotid artery stenting and endarterectomy, and adversely affects long-term prognosis [4, 5]. Accurate evaluation of coronary status prior to cerebrovascular angiography or intervention is therefore essential in this population [6]. Nevertheless, the routine application of coronary computed tomography angiography (CCTA) or invasive coronary angiography for preoperative screening is constrained by several factors, including high cost, radiation exposure, and procedural invasiveness, which limit their universal implementation. Existing clinical scoring systems derived from conventional risk factors or circulating biomarkers offer limited predictive specificity and are inadequate for precise individualized risk stratification [7, 8]. Although ultrasound-derived carotid intima-media thickness and plaque burden are associated with CAD, their predictive performance remains suboptimal, and reproducibility is variable [9]. Consequently, there is a pressing clinical need for an efficient, non-invasive risk-assessment approach that can be readily integrated into existing diagnostic workflows.

Carotid CTA is a widely used imaging modality for evaluating carotid artery stenosis. Conventional evaluation focuses primarily on the degree of luminal stenosis, whereas quantitative analysis of plaque components (e.g., calcified and lipid volumes) can more accurately reflect plaque vulnerability and atherosclerotic burden [10]. However, manual quantification is time-consuming and labor-intensive and has poor reproducibility, which limits its clinical translation. Recently, deep-learning–based automated plaque analysis has enabled rapid and accurate plaque segmentation and component quantification on CCTA images [11]. We hypothesized that a diagnostic model built upon automated quantification of carotid plaque components on routine carotid CTA could identify coexisting obstructive coronary artery disease (OCAD) in patients with carotid stenosis, thus providing a novel and efficient risk stratification tool.

Therefore, this study retrospectively included a large cohort of patients who underwent both carotid and coronary CTA within a 3-month interval. The primary aim was to develop and validate a diagnostic model for OCAD based on automated carotid plaque metrics in a well-defined dual-center cohort, and to systematically assess its independent diagnostic performance.

## Materials and methods

### Study population

This retrospective, dual-center cohort study aimed to develop and validate a diagnostic model for OCAD using carotid plaque characteristics. We consecutively enrolled adult patients (aged ≥ 18 years) who underwent carotid CTA at the participating hospitals between January 2011 and June 2025, and who subsequently underwent CCTA within 3 months. Exclusion criteria were: (1) occlusion of the common or internal carotid artery; (2) prior carotid or coronary revascularization (stenting or bypass grafting); (3) incomplete clinical or imaging data; and (4) active malignancy or end-stage renal disease.

The predefined cohort structure was as follows: patients were divided chronologically into a modeling cohort (Cohort 1, from January 2011 to December 2023) for model development and internal validation, and a temporally independent external validation cohort (Cohort 2, from January 2024 to June 2025) for assessing generalizability. Within Cohort 1, patients were randomly divided into the training and the internal validation sets at a 7:3 ratio. This retrospective observational study received approval from the Institutional Review Board of Nanjing Jinling Hospital (approval no. 2023DZKY-019-03) on March 19, 2023, with informed consent waived due to its retrospective design.

Demographics, cardiovascular risk factors, medication use, and laboratory results were extracted from the electronic medical record system. Key variables were defined as follows. Hypertension: systolic blood pressure ≥ 140 mmHg, diastolic blood pressure ≥ 90 mmHg, or current antihypertensive treatment. Diabetes: Fasting glucose ≥ 7.0 mmol/L, glycated hemoglobin A_1_c ≥ 6.5%, or a documented diagnosis treated with glucose-lowering therapy. Dyslipidemia: low-density lipoprotein cholesterol (LDL-C) ≥ 3.4 mmol/L, high-density lipoprotein cholesterol (HDL-C) < 1.0 mmol/L (men) or < 1.3 mmol/L (women), triglycerides ≥ 1.7 mmol/L, or current lipid-lowering treatment. Smoking History: Documented former or current smoking.

### Study outcomes

The primary outcome was OCAD, defined as a luminal stenosis ≥ 50% in at least one major epicardial coronary artery (left main, left anterior descending, left circumflex, or right coronary artery) on CCTA. The secondary outcome was severe coronary stenosis, defined as ≥ 70% luminal stenosis in any major coronary artery.

### Carotid and coronary CTA image acquisition and analysis

#### Image acquisition

All scans were performed on a dual-source CT scanner (Somatom Definition Flash, Siemens Healthineers). For CCTA, the scan range extended from 1 cm below the carina to 1 cm below the diaphragm. For carotid CTA, images were acquired in a caudocranial direction from the aortic arch to the skull base. A bolus of 60–80 mL of iodinated contrast medium (Iopromide, 300–370 mg I/mL, Bayer) was injected via an antecubital vein at a flow rate of 4–5 mL/s, followed by a 40-mL saline flush at the same rate. For CCTA, acquisition used prospective electrocardiogram triggering (30%–80% of the R-R interval) with the following parameters: tube voltage 100–120 kV, automatic current modulation, slice thickness 1 mm, slice increment 1 mm, rotation time 0.28 s/r, collimation 128 × 0.6 mm, pitch 1.2, matrix 512 × 512, temporal resolution 75 ms. For carotid CTA, a bolus-tracking technique was used with the region of interest placed in the descending aorta and a trigger threshold of 100 Hounsfield units (HU); scanning commenced 3–4 s after the threshold was reached. Acquisition parameters were as follows: tube voltage 120 kV, tube current 200–350 mA, slice thickness 1 mm, slice increment 1 mm, matrix 512 × 512.

#### Image analysis

Stenosis and plaque analysis were performed using artificial intelligence (AI)-assisted software (uAI Coronary, uAI Discover Cerebral & Carotid Vessels, United Imaging Intelligence). The accuracy and consistency of this software have been validated previously [12]. This software enables fully automated analysis of CTA images, encompassing vascular structure segmentation, plaque detection and quantification, and measurement and grading of stenosis. First, the coronary artery tree (vessels ≥ 2 mm in diameter) and carotid arteries were automatically segmented from the original DICOM data using deep learning-based models and reconstructed into three-dimensional images. Next, the system detected and quantified stenotic lesions and plaque components, and generated a preliminary report detailing their location and severity. For coronary arteries, the stenosis severity was graded according to the Coronary Artery Disease Reporting and Data System (CAD-RADS) [13]. For carotid arteries, quantitative parameters mainly included plaque morphological parameters (such as maximum lumen stenosis ratio and remodeling index) and the absolute volume and relative proportion of each plaque component (lipid, fibrotic, and calcified). The software performed fully automated analyses on all enrolled cases. Subsequently, a senior cardiovascular radiologist (with ≥ 10 years of CTA experience) conducted a quality control review of cases in which automated processing failed. All included cases underwent automated analysis. After exclusion based on the prespecified study criteria, 19 cases were excluded because poor image quality and severe motion artifacts resulted in inaccurate automated analysis results. Therefore, for all cases ultimately included, automated quantitative plaque metrics were derived directly from the original automated output.

### Statistical analysis

All statistical analyses were performed using SPSS (version 26.0, IBM) and R software. Continuous variables are presented as medians with interquartile ranges (IQR), and categorical variables as frequencies (*n*) and percentages. For regression modeling, carotid plaque volume metrics were transformed using the Box–Cox method to reduce skewness and approximate normal distributions. Descriptive comparisons are reported using the original scale unless otherwise specified. Group comparisons were performed using the independent samples *t*-test or Mann–Whitney *U*-test for continuous variables, and the Pearson chi-square test or Fisher’s exact test for categorical variables, as appropriate.

Univariable and multivariable logistic regression analyses were performed to examine associations between variables and outcomes, calculating odds ratios (OR) with 95% confidence intervals (CI). To develop a parsimonious diagnostic model, variable selection was performed using least absolute shrinkage and selection operator (LASSO) regression with 10-fold cross-validation in the training set. The optimal penalty parameter (λ) was selected according to the “one-standard-error” rule (λ.1se). Variance inflation factors (VIF) were calculated to assess multicollinearity; all VIF values were < 5, confirming the absence of significant multicollinearity.

Model performance was assessed using the area under the receiver operating characteristic curve (AUC), sensitivity, specificity, and accuracy. The discriminative abilities of the models were compared using the DeLong test. Model validation included internal validation and temporally independent external validation. Calibration was assessed visually using calibration curves. Decision curve analysis (DCA) was performed to quantify the net clinical benefit across a range of probability thresholds.

A sensitivity analysis was performed using severe coronary stenosis (≥ 70%) as the alternative outcome.

A two-sided *p*-value < 0.05 was considered to indicate statistical significance.

## Results

### Study population

**Fig. 1:**
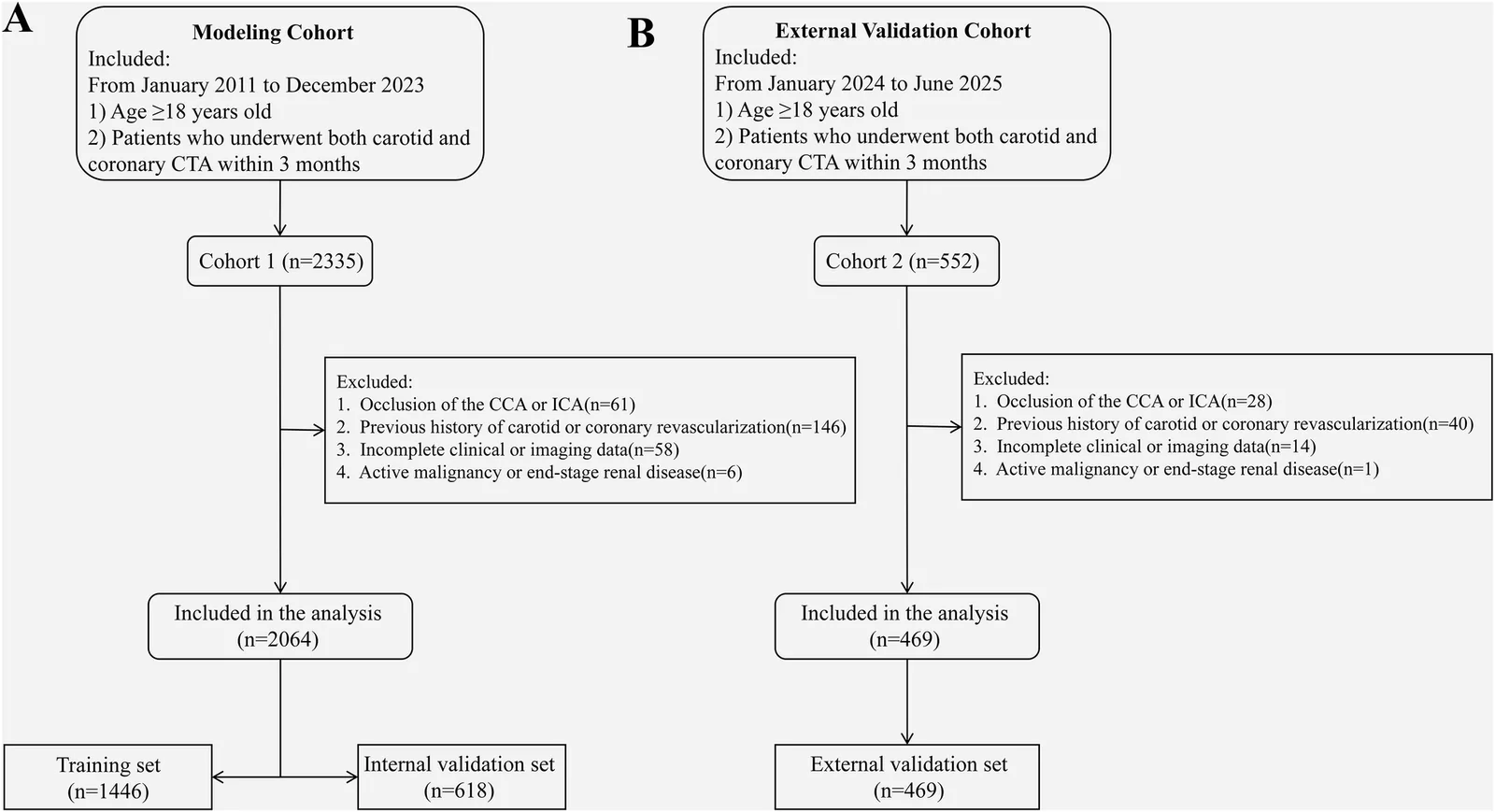
Flowchart of study population inclusion and exclusion. **A** Cohort 1 was used as the modeling cohort, and (**B**) Cohort 2 as the external validation cohort. CCA, common carotid artery; ICA, internal carotid artery; CTA, computed tomography angiography

A total of 2887 consecutive patients were initially screened. After excluding 354 patients who met predefined exclusion criteria (Fig. 1), 2533 patients were ultimately enrolled. Of these, 2064 were assigned to the modeling cohort and 469 to the temporally independent external validation cohort; within the modeling cohort, 1446 constituted the training set and 618 the internal validation set.

In the modeling cohort, the median age was 64.0 years (IQR, 56.0–72.0), and 36.7% of patients were female. The prevalence of hypertension, diabetes, and dyslipidemia was 68.7%, 30.4%, and 83.5%, respectively, and 75.4% of patients were on lipid-lowering therapy. Key laboratory values are summarized in Table 1.

**Table 1:** Baseline variables of the study population by OCAD status

Variables	Overall (*n* = 2064)	Without OCAD (*n* = 1343)	With OCAD (*n* = 721)	*p*-value
Demographics				
Female sex, *n* (%)	757 (36.68%)	546 (40.66%)	211 (29.26%)	< 0.001
Age, years	64.00 (56.00–72.00)	61.00 (53.00–69.00)	68.00 (61.00–74.00)	< 0.001
BMI, kg/m²	24.57 (22.43–26.83)	24.57 (22.25–26.98)	24.60 (22.50–26.65)	0.900
Cardiovascular risk factors				
Hypertension, *n* (%)	1327 (68.72%)	779 (62.77%)	548 (79.42%)	< 0.001
Diabetes, *n* (%)	581 (30.43%)	281 (23.03%)	300 (43.54%)	< 0.001
Smoking history, *n* (%)	644 (34.38%)	341 (29.55%)	303 (42.14%)	< 0.001
Cholesterol-lowering drugs, *n* (%)	1419 (75.44%)	813 (67.47%)	606 (89.64%)	< 0.001
Dyslipidemia, *n* (%)	1575 (83.47%)	936 (77.74%)	639 (93.56%)	< 0.001
Laboratory indicators				
Glucose, mmol/L	5.10 (4.60–6.00)	5.00 (4.50–5.70)	5.20 (4.70–6.70)	< 0.001
Total cholesterol, mmol/L	4.15 (3.45–4.86)	4.21 (3.51–4.88)	3.97 (3.34–4.86)	0.143
Triglycerides, mmol/L	1.32 (0.96–1.86)	1.31 (0.97–1.88)	1.32 (0.94–1.82)	0.971
HDL-C, mmol/L	1.07 (0.90–1.26)	1.09 (0.93–1.29)	1.02 (0.87–1.21)	< 0.001
LDL-C, mmol/L	2.35 (1.78–2.93)	2.40 (1.83–2.92)	2.22 (1.70–2.93)	0.371
C-reactive protein, mg/L	1.10 (0.50–3.10)	1.00 (0.50–3.10)	1.10 (0.50–3.50)	0.161
Carotid plaque composition				
Carotid stenosis severity	0.42 (0.30–0.56)	0.36 (0.25–0.48)	0.49 (0.38–0.66)	< 0.001
Calcified volume	2.64 (1.43–3.50)	1.96 (1.43–3.00)	3.49 (2.77–3.88)	< 0.001
Fibrotic volume	1.54 (1.25–2.29)	1.25 (1.25–2.04)	2.20 (1.41–2.47)	< 0.001
Lipid volume	1.70 (1.21–2.18)	1.29 (1.21–2.00)	2.14 (1.60–2.30)	< 0.001

Continuous variables are presented as median (interquartile range, Q1–Q3) owing to non-normal distributions and were compared using the Mann–Whitney *U*-test. Categorical variables are presented as frequencies (*n*, %) and were compared using the Pearson chi-square test. Plaque volumes were transformed using the Box–Cox method for regression modeling, whereas descriptive statistics are presented on the original scale unless otherwise specified

*BMI* body mass index, *HDL-C* high-density lipoprotein cholesterol, *LDL-C*, low-density lipoprotein cholesterol

Baseline characteristics of the modeling cohort, stratified by OCAD status, are presented in Table 1. Compared with patients without OCAD, those with OCAD were significantly older, less likely to be female, and had a higher prevalence of all conventional cardiovascular risk factors—including hypertension, diabetes, dyslipidemia, and smoking history (all *p* < 0.001). With regard to biochemical markers, patients with OCAD also showed significantly higher fasting glucose and lower HDL-C levels (both *p* < 0.001). Critically, all AI-quantified carotid plaque component volumes (calcified, fibrotic, and lipid) and carotid stenosis severity were substantially greater in patients with OCAD (all *p* < 0.001). No significant differences were observed between groups for BMI, total cholesterol, triglycerides, LDL-C, or C-reactive protein.

A comparison of baseline characteristics between the modeling cohort and the external validation cohort is presented in Table S1.

### Univariable and multivariable associations with OCAD

Univariable analysis revealed that most variables were significantly associated with OCAD (*p* < 0.001) (Table 2). Female sex, advanced age, hypertension, diabetes, smoking history, use of cholesterol-lowering drugs, dyslipidemia, elevated glucose, and lower HDL-C were all significantly associated with OCAD. Carotid stenosis severity also showed a strong positive association (OR, 16.48; 95% CI: 10.33–26.29; *p* < 0.001). Calcified, fibrotic, and lipid carotid plaque volumes showed positive associations (OR, 3.25, 4.49, and 6.47, respectively; *p* < 0.001). These plaque volume metrics and carotid stenosis severity demonstrated notable stand-alone diagnostic accuracy in the training set, with AUCs of 0.781, 0.717, 0.737, and 0.689, respectively (Table 3).

**Table 2:** Univariable and multivariable logistic regression analyses of OCAD-related variables

Variables	Univariable		Multivariable	
	OR (95% CI)	*p*-value	OR (95% CI)	*p*-value
Clinical characteristics				
Female sex	0.60 (0.50–0.73)	< 0.001	0.71 (0.50–1.01)	0.054
Age, years	1.06 (1.05–1.07)	< 0.001	1.01 (0.99–1.02)	0.342
BMI, kg/m²	1.00 (0.97–1.03)	0.900	1.00 (0.96–1.04)	0.918
Cardiovascular risk factors				
Hypertension	2.29 (1.84–2.84)	< 0.001	0.98 (0.69–1.38)	0.909
Diabetes	2.58 (2.11–3.15)	< 0.001	1.77 (1.25–2.49)	0.001
Smoking history	1.74 (1.43–2.11)	< 0.001	1.02 (0.74–1.40)	0.925
Cholesterol-lowering drugs	4.17 (3.17–5.50)	< 0.001	2.44 (1.32–4.52)	0.005
Dyslipidemia	4.16 (2.98–5.81)	< 0.001	1.00 (0.42–2.34)	0.991
Laboratory indicators				
Glucose, mmol/L	1.21 (1.15–1.28)	< 0.001	1.03 (0.94–1.13)	0.500
Total cholesterol, mmol/L	0.93 (0.85–1.02)	0.144	1.90 (1.16–3.10)	0.011
Triglycerides, mmol/L	1.00 (0.91–1.09)	0.971	0.81 (0.65–1.01)	0.067
HDL-C, mmol/L	0.39 (0.27–0.57)	< 0.001	0.18 (0.08–0.39)	< 0.001
LDL-C, mmol/L	0.95 (0.85–1.06)	0.371	0.61 (0.37–1.03)	0.064
C-reactive protein, mg/L	1.00 (1.00–1.01)	0.165	1.02 (1.00–1.03)	0.027
Carotid plaque composition				
Carotid stenosis severity	16.48 (10.33–26.29)	< 0.001	1.71 (0.80–3.64)	0.166
Calcified volume	3.25 (2.89–3.65)	< 0.001	2.43 (1.95–3.04)	< 0.001
Fibrotic volume	4.49 (3.73–5.39)	< 0.001	1.37 (1.01–1.85)	0.046
Lipid volume	6.47 (5.20–8.05)	< 0.001	1.26 (0.85–1.87)	0.256

*OR* odds ratio, *CI* confidence interval, *BMI* body mass index, *HDL-C* high-density lipoprotein cholesterol, *LDL-C* low-density lipoprotein cholesterol

**Table 3:** Diagnostic performance of different models for OCAD

Dataset/variables	AUC (95% CI)	Sensitivity (95% CI, %)	Specificity (95% CI, %)	Accuracy (95% CI, %)
Single variables				
Male sex	–	29.3 (26.1–32.7)	59.3 (56.7–62.0)	48.8 (46.7–51.0)
Age, years	0.669 (0.639–0.694)	70.3 (68.1–71.6)	54.0 (51.9–54.6)	59.7 (58.4–60.5)
Diabetes	–	43.5 (39.9–47.3)	77.0 (74.5–79.3)	64.9 (62.7–67.0)
Cholesterol-lowering drugs	–	89.6 (87.1–91.7)	32.5 (29.9–35.2)	53.1 (50.8–55.3)
Carotid stenosis severity	0.689 (0.665–0.716)	73.8 (70.3–76.9)	56.1 (52.9–59.3)	63.7 (61.3–66.0)
Calcified volume	0.781 (0.756–0.805)	70.7 (67.6–71.0)	74.7 (73.0–75.4)	73.3 (71.9–73.8)
Fibrotic volume	0.717 (0.682–0.745)	74.7 (73.0–76.3)	59.7 (56.7–60.4)	64.9 (63.2–65.3)
Lipid volume	0.737 (0.707–0.764)	54.5 (53.6–57.3)	82.8 (78.7–83.5)	72.2 (70.0–73.1)
Training set				
Clinical model	0.723 (0.696–0.749)	36.2 (32.2–40.5)	85.8 (83.4–87.9)	68.5 (66.0–70.9)
Plaque model	0.785 (0.761–0.810)	56.8 (52.4–61.2)	83.3 (80.8–85.6)	74.1 (71.7–76.3)
Integrated model	0.800 (0.777–0.824)	57.6 (53.3–61.9)	83.5 (81.0–85.8)	74.5 (72.2–76.7)
Internal validation set				
Clinical model	0.752 (0.713–0.791)	41.2 (34.9–47.9)	86.1 (82.4–89.1)	70.4 (66.6–74.0)
Plaque model	0.824 (0.789–0.858)	61.6 (54.9–67.9)	84.8 (80.9–88.2)	76.7 (73.2–80.0)
Integrated model	0.840 (0.808–0.872)	66.7 (60.1–72.6)	85.1 (81.3–88.2)	78.6 (75.2–81.8)
Modeling cohort				
Clinical model	0.678 (0.651–0.704)	71.6 (68.2–74.7)	54.6 (51.9–57.2)	60.5 (58.4–62.6)
Plaque model	0.800 (0.781–0.819)	74.5 (71.2–77.5)	74.5 (72.1–76.7)	74.5 (72.5–76.3)
Integrated model	0.797 (0.779–0.816)	76.0 (72.8–79.0)	71.7 (69.2–74.1)	73.2 (71.3–75.1)
External validation cohort				
Clinical model	0.660 (0.615–0.710)	62.9 (55.4–69.9)	62.9 (57.1–68.3)	62.9 (58.6–67.0)
Plaque model	0.813 (0.773–0.857)	71.2 (63.9–77.6)	77.3 (72.1–81.8)	75.0 (70.9–78.8)
Integrated model	0.805 (0.765–0.846)	74.1 (67.0–80.2)	73.9 (68.5–78.8)	74.0 (69.8–77.8)

The modeling cohort (from January 2011 to December 2023) was randomly divided into training and internal validation sets at a 7:3 ratio; the external validation cohort was temporally independent (from January 2024 to June 2025). AUC, area under the curve; Clinical Model: male sex, age, diabetes, and cholesterol-lowering drugs; Plaque Model: calcified volume, fibrotic volume, and lipid volume; Integrated Model: combination of clinical and plaque model variables. AUC was not calculated for single binary variables (e.g., sex, diabetes)

**Fig. 2:**
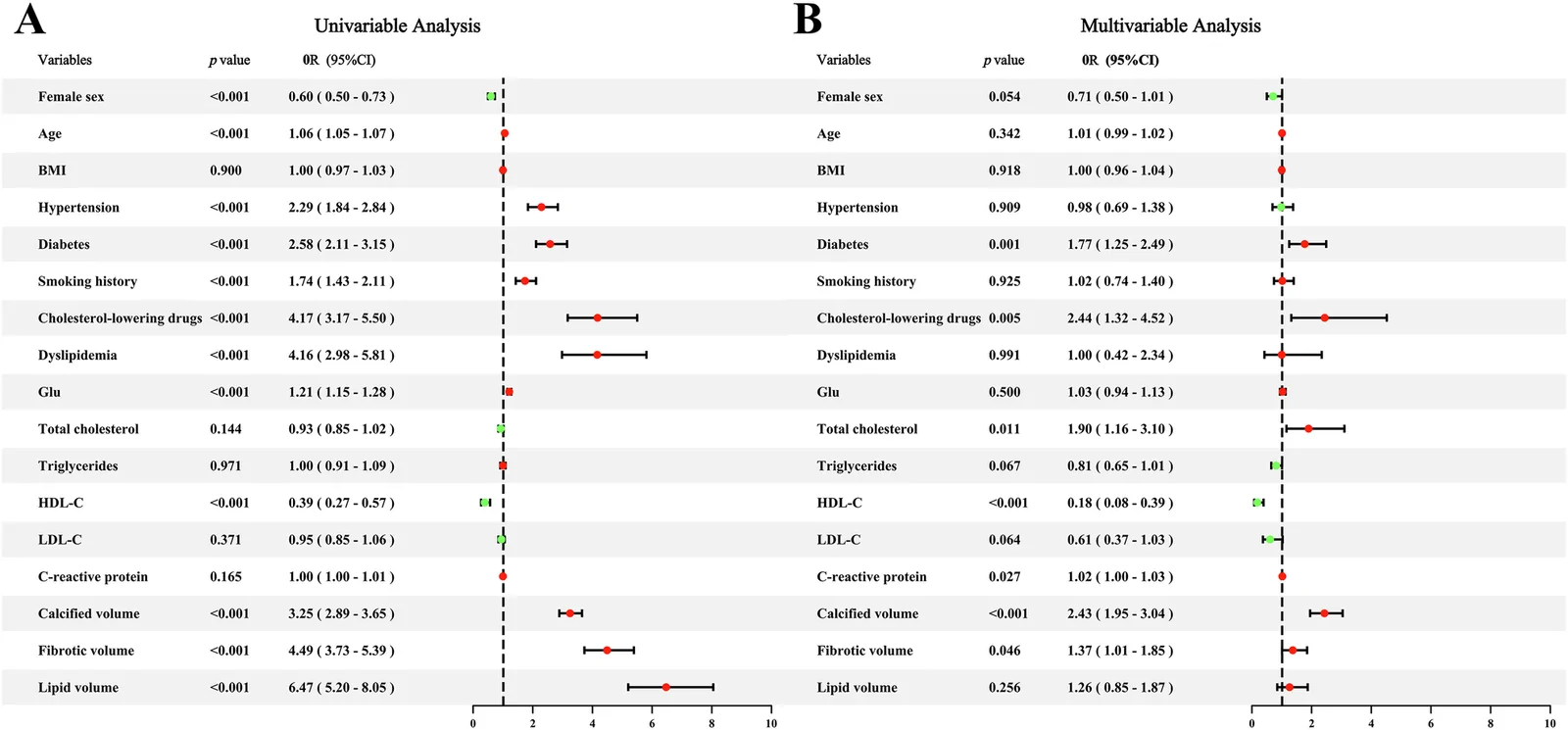
Forest plot of univariable (**A**) and multivariable analyses (**B**). Dots represent odds ratios (OR); horizontal lines represent 95% confidence intervals (CI). The vertical dashed line indicates OR = 1 (no effect). BMI, body mass index; HDL-C, high-density lipoprotein cholesterol; LDL-C, low-density lipoprotein cholesterol; Glu, glucose

Multivariable analysis identified HDL-C as an independent protective factor (OR, 0.18; 95% CI: 0.08–0.39; *p* < 0.001). Diabetes (OR, 1.77; 95% CI: 1.25–2.49; *p* = 0.001), use of cholesterol-lowering drugs (OR, 2.44; 95% CI: 1.32–4.52; *p* = 0.005), elevated total cholesterol (OR, 1.90; 95% CI: 1.16–3.10; *p* = 0.011), elevated C-reactive protein (OR, 1.02; 95% CI: 1.00–1.03; *p* = 0.027), greater calcified plaque volume (OR, 2.43; 95% CI: 1.95–3.04; *p* < 0.001), and greater fibrotic plaque volume (OR, 1.37; 95% CI: 1.01–1.85; *p* = 0.046) were identified as independent risk factors for OCAD. These associations are summarized graphically in the forest plot (Fig. 2).

### Development and validation of the models

#### Variable selection

To develop parsimonious models and avoid information leakage, LASSO regression with 10-fold cross-validation was applied only to the training set. Using the λ.1se criterion (λ = 0.0269) to balance complexity and accuracy, seven predictors were retained: sex, age, diabetes, use of cholesterol-lowering drugs, calcified plaque volume, fibrotic plaque volume, and lipid plaque volume (Fig. S1).

To assess the incremental value of carotid plaque quantification over routine clinical data, three logistic-regression models were constructed. Clinical model: included only the clinical variables identified by LASSO (sex, age, diabetes, and use of cholesterol-lowering drugs). Plaque model: included only the three quantitative plaque components (calcified, fibrotic, and lipid volumes). Integrated model: combined all variables from the clinical and plaque models. Multicollinearity was assessed and was acceptable for all variables (all VIF < 3, Table S2).

### Model performance and validation

**Fig. 3:**
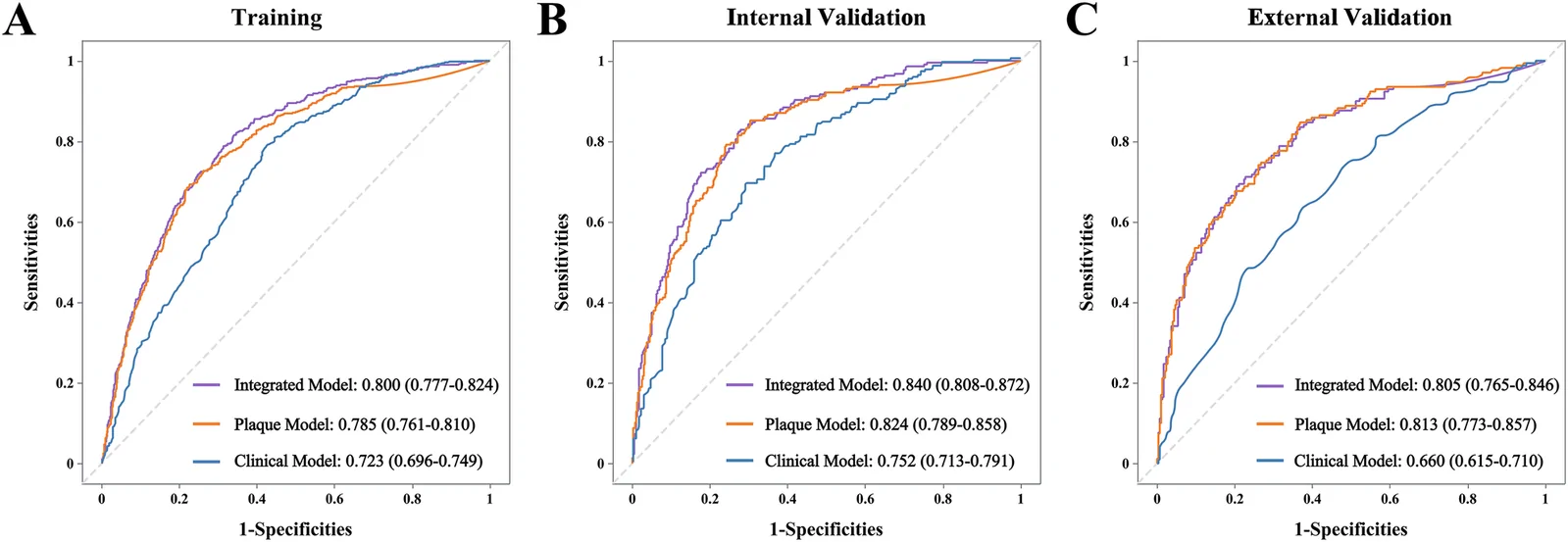
ROC Curves Comparing Three Models Across Different Datasets. **A** Training set. **B** Internal validation set. **C** External validation set. The blue, orange, and purple curves represent the Clinical, Plaque, and Integrated models, respectively. AUC, area under the curve; Clinical Model: male sex, age, diabetes, and cholesterol-lowering drugs; Plaque Model: calcified volume, fibrotic volume, and lipid volume; Integrated Model: combination of clinical and plaque model variables

The diagnostic performance of the three models in the training, internal validation, and external validation sets is summarized in Table 3. In the training set, the integrated model achieved an AUC of 0.800 (95% CI: 0.777–0.824), with sensitivity of 57.6% and specificity of 83.5%. Performance was consistent in the internal validation set (AUC, 0.840; 95% CI: 0.808–0.872; sensitivity, 66.7%; specificity, 85.1%) and in the external validation cohort (AUC, 0.805; 95% CI: 0.765–0.846; sensitivity, 74.1%; specificity, 73.9%). A comparison of the receiver operating characteristic (ROC) curves of the three models across datasets is shown in Fig. 3.

**Fig. 4:**
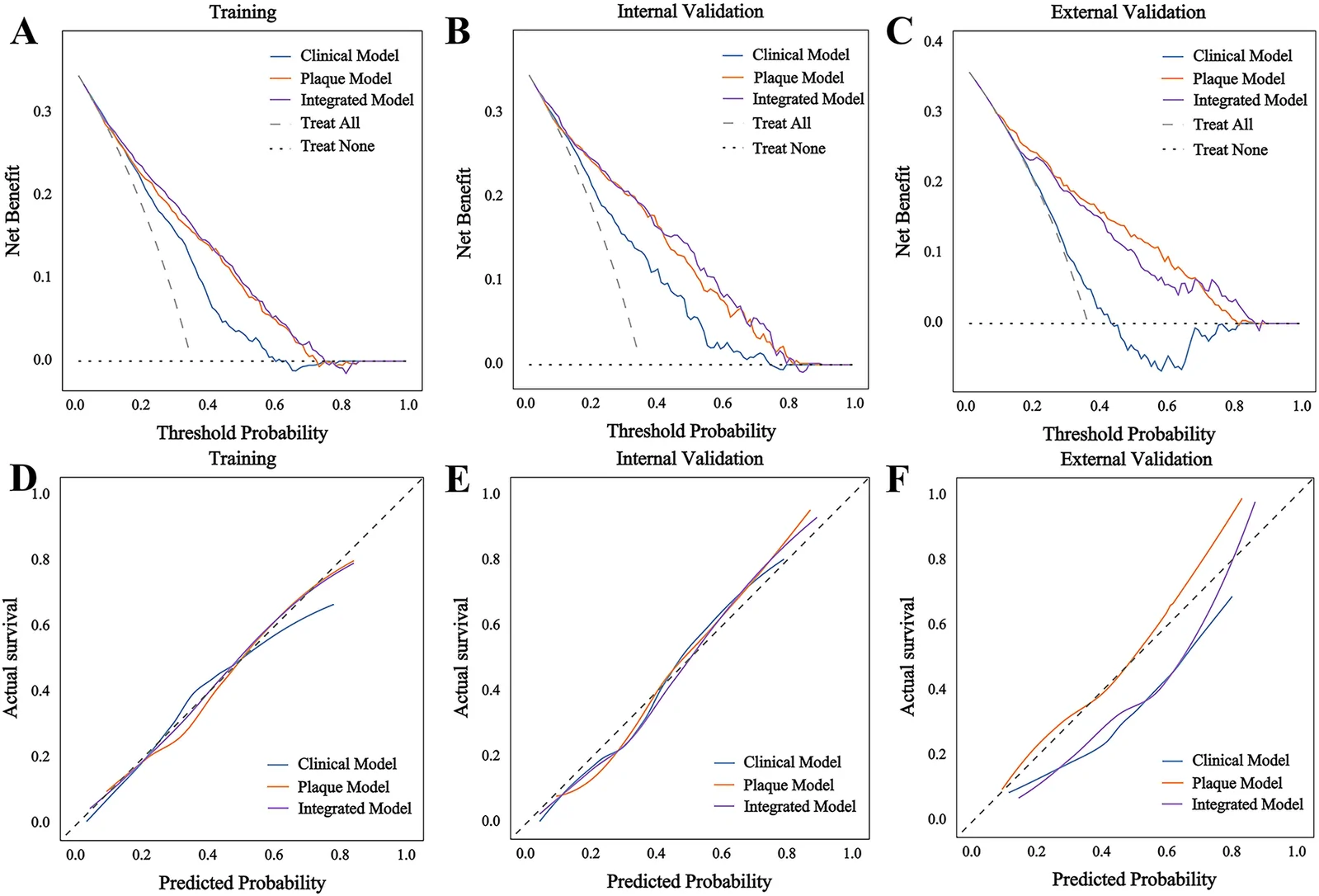
DCA and calibration curves comparing three models across different datasets. **A**–**C** DCA for the training set (**A**), internal validation set (**B**), and external validation set (**C**). The blue, orange, and purple curves represent the Clinical, Plaque, and Integrated models, respectively. The gray long dashed line (all) indicates that all patients received the intervention; the black short dashed line (none) shows that no patients received the intervention (net benefit = 0). **D**–**F** Calibration curves for the training set (**D**), internal validation set (**E**), and external validation set (**F**). The diagonal dashed line represents ideal calibration (predicted probability = observed outcome)

DCA showed that, across a wide range of threshold probabilities, the clinical net benefit of both the plaque model and the integrated model was significantly greater than that of the clinical model (Fig. 4A–C). The calibration curves demonstrated good agreement between the predicted probabilities and the observed outcomes for the integrated model across datasets (Fig. 4D–F).

In each dataset, the DeLong test showed that the plaque model and the integrated model significantly outperformed the clinical model (all *p* < 0.001) (Table S3). Notably, the discriminative ability of the plaque model and the integrated model was not significantly different in both the modeling cohort (AUC, 0.800 vs 0.797, *p* = 0.124) and the external validation cohort (AUC, 0.813 vs 0.805; *p* = 0.809), indicating that a model based solely on automated carotid plaque quantification achieves comparable performance to the full integrated model.

### Clinical application as a risk stratification tool

**Fig. 5:**
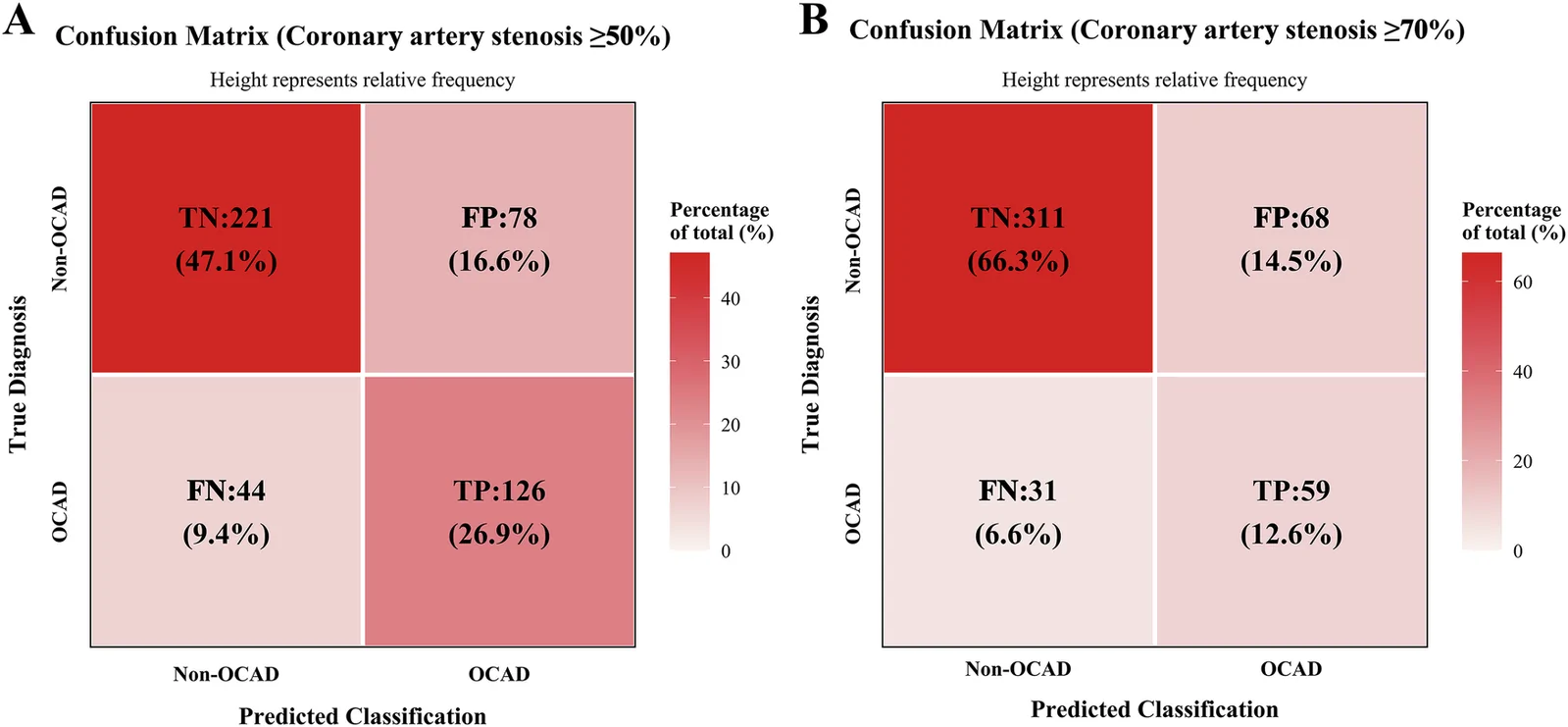
Confusion matrix for the integrated model in the external validation cohort. **A** Results when coronary artery stenosis ≥ 50% is the diagnostic threshold. **B** Corresponding results at the ≥ 70% stenosis threshold. The matrices show the distribution of predicted vs actual diagnoses for the integrated model in the external validation cohort (*n* = 469). Column heights represent relative frequencies as a percentage of the total. TN, true negative; FP, false positive; FN, false negative; TP, true positive

When applied as a risk stratification tool in the external validation cohort (*n* = 469) at the optimal cut-off, the integrated model classified 56.5% of patients as low risk. This strategy maintained a high negative predictive value (83.4%) while identifying 74.1% of true OCAD cases (Fig. 5).

**Fig. 6:**
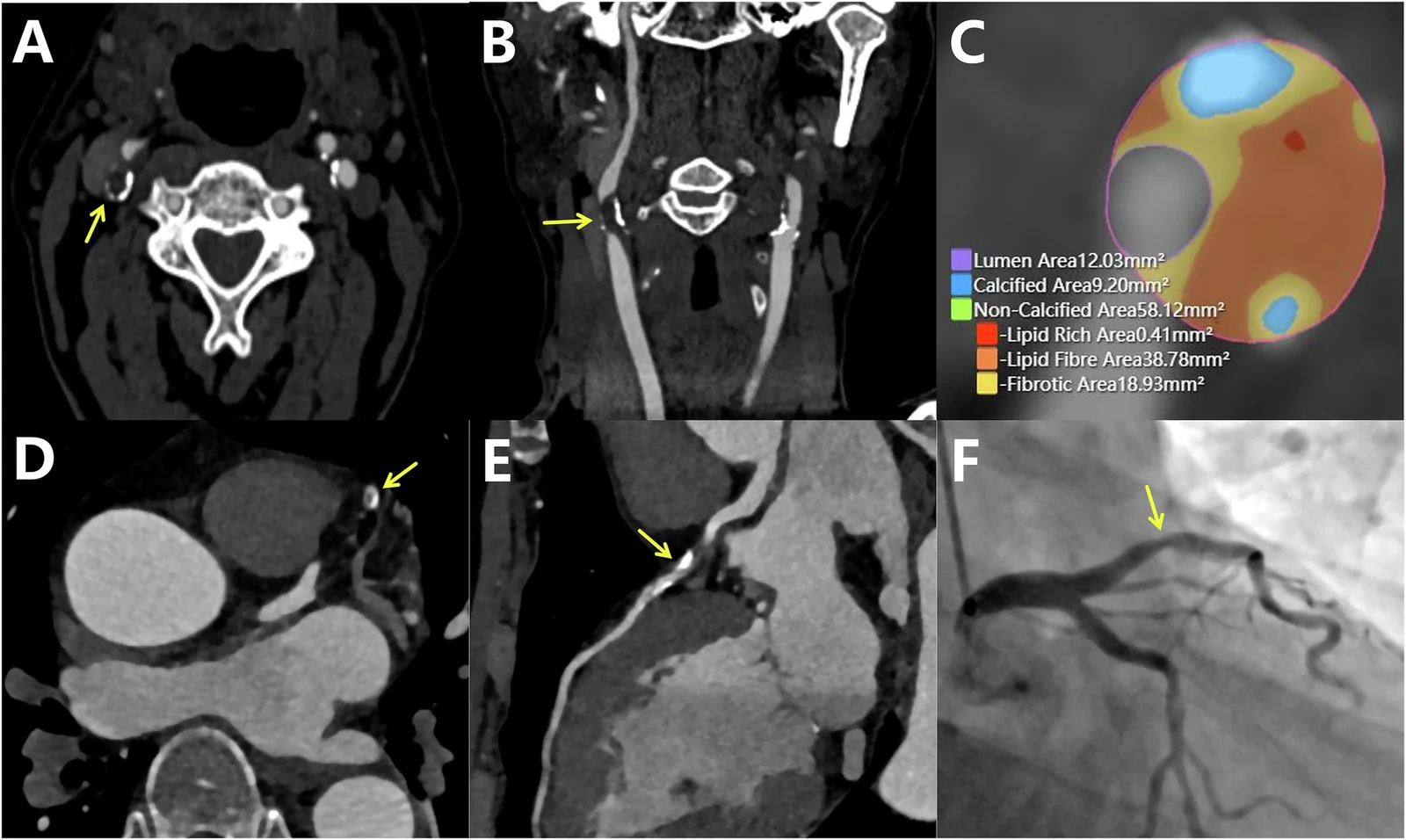
Representative case illustrating quantitative carotid plaque analysis and its correlation with obstructive CAD. **A** Axial carotid CTA image, showing severe (approximately 80%) luminal stenosis caused by a mixed plaque (yellow arrow) in the C1 segment of the right internal carotid artery. **B** Three-dimensional volume-rendered reconstruction image of carotid CTA showing the right internal carotid artery in full and the C1-segment stenosis (yellow arrow). **C** Vascular plaque quantification software interface, with automatic measurement and display of plaque-component volumes (lipid and calcified components). **D** Axial coronary CTA image from the same patient, showing a mixed plaque (yellow arrow) in the proximal segment of the left anterior descending artery causing moderate luminal stenosis (approximately 60%). **E** Three-dimensional volume-rendered reconstruction of CCTA, showing the course of the left anterior descending artery (LAD) and the proximal stenosis (yellow arrow). **F** Invasive coronary angiography of the same patient confirming moderate stenosis in the proximal segment of the LAD (yellow arrow). Yellow arrows indicate the locations of plaques/stenoses

Figure 6 illustrates a representative case of a patient with OCAD. The automated plaque segmentation and quantitative compositional analysis on the carotid CTA images demonstrate an intuitive correlation with the significant vessel stenosis confirmed by CCTA.

### Sensitivity analysis

To test the robustness of the findings, the analysis was repeated using severe coronary stenosis ( ≥ 70%) as the outcome. The results were consistent with those of the main analysis. Detailed results are provided in the supplementary material (Tables S4–6 and Figs. S2 and S3). In the external validation cohort, the model incorporating only carotid plaque metrics achieved an AUC of 0.801 (95% CI: 0.755–0.846), comparable to the performance of the full integrated model (AUC, 0.802; 95% CI: 0.753–0.847) and substantially superior to that of the clinical model (AUC, 0.623; 95% CI: 0.567–0.686). This pattern was maintained across the training and internal validation sets.

## Discussion

By analyzing a large cohort with paired carotid and coronary CTA examinations, the present study confirmed that the automated quantitative measures of carotid plaque can be used to construct a robust diagnostic model that effectively identifies patients with OCAD. In each dataset, the plaque and integrated models showed similar performance, and both significantly outperformed the clinical model (*p* < 0.001). In the temporally independent external validation cohort, the proposed model demonstrated excellent generalizability (AUC, 0.805) and had clear clinical triage value: the model reliably identified 74.1% of OCAD cases and classified 56.5% of patients as low risk. Among patients who have undergone carotid CTA, the model may therefore serve as an adjunctive risk-stratification tool for OCAD and help to optimize clinical decision-making.

The diagnostic performance of the model based only on clinical indicators was weaker than that of the plaque-based model and the integrated model, and the AUC value in the training set was 0.723. This finding is consistent with the limited performance of established cardiovascular risk scores. Traditional risk factors indicate upstream susceptibility to atherosclerosis, but do not directly quantify disease burden. In contrast, quantitative carotid plaque volume provides an imaging-based measure of systemic atherosclerotic burden and reflects pathological features shared with coronary disease, such as lipid deposition, fibrosis, and calcification. These characteristics may underlie the superior diagnostic value of plaque metrics over clinical variables for identifying OCAD. It is worth noting that the plaque-only model performed comparably to the integrated model in the external cohort. This may be because the quantitative plaque parameters inherently reflect the cumulative pathological effects of long-term exposure to clinical risk factors, such that clinical variables did not provide significant incremental information. In addition, the objectivity and standardization of imaging-based quantitative metrics are superior to those of retrospective clinical data, which may confer greater robustness across different populations.

In this study, automatically extracted carotid plaque parameters—particularly calcified plaque volume and fibrotic plaque volume—were independent risk factors for OCAD (adjusted OR, 2.43 and 1.37, respectively). Although lipid plaque volume was selected by LASSO, it was not an independent predictor in the multivariable model, possibly because of shared variance among plaque components or because calcified and fibrotic volumes better reflected cumulative atherosclerotic burden in this cohort. This supports the view that atherosclerosis is a systemic disease in which key pathological processes—including inflammation, lipid deposition, fibrosis, and calcification—evolve concurrently in the carotid and coronary arteries, rendering carotid plaque composition and burden a candidate imaging biomarker of CAD [14]. Previous studies relied largely on carotid ultrasound to evaluate the association of carotid disease with coronary heart disease. Conventional ultrasonographic morphological metrics (maximum plaque length, area, and intima-media thickness) have been shown to be effective predictors [15–18]. For example, the maximum plaque length showed good diagnostic performance for identifying the presence and severity of CAD (AUC, 0.767) [15]. Contrast-enhanced ultrasound, which visualizes intraplaque neovascularization following intravenous administration of a microbubble contrast agent, has further improved the prediction of OCAD [19]. However, these techniques remain operator-dependent, constrained by their two-dimensional nature, or technically demanding. Herr et al used ultrasound grayscale pixel-distribution analysis to quantify the calcified, fibrotic, and lipid components of carotid plaque in 468 patients; the calcified-tissue proportion was independently associated with CAD severity [20]. In contrast, studies of automated plaque quantification using carotid CTA are scarce. The automated technique used in this study enables objective assessment of the calcified, fibrotic, and lipid components of carotid plaques. Our analyses showed that these quantitative plaque volume metrics, such as calcified volume, showed good diagnostic performance on their own (AUC, 0.781). These findings highlight the potential of using automated quantitative analysis of existing carotid CTA images as an adjunctive risk-stratification tool for coronary heart disease risk assessment.

The carotid plaque quantification model demonstrated excellent and robust diagnostic performance in external validation (AUC, 0.813), suggesting its potential utility for identifying patients with OCAD. The primary purpose of conventional cardiovascular risk stratification tools, such as the Framingham score, which predicts the 10-year risk of cardiovascular events, and the coronary artery calcium (CAC) score, which quantifies the burden of coronary artery calcification, is to assess the long-term risk or the burden of specific plaque components, rather than to identify current OCAD [21, 22]. Some studies have attempted to modify traditional models. For example, Chen et al [23] developed a modified Framingham score to predict OCAD in 1,262 patients undergoing elective coronary angiography. The model included variables such as anemia, high-sensitivity C-reactive protein, and left ventricular ejection fraction and achieved an AUC of 0.703 (95% CI: 0.69–0.77). Moreover, the model’s clinical utility is limited by its development in a single-center study without external validation. The model developed in the present study is not intended to replace these risk assessment tools; rather, it is designed to assist in identifying individuals needing further coronary evaluation among patients who have undergone carotid CTA for cerebrovascular indications. Our results support the effectiveness of this strategy: when applied as an adjunctive risk stratification tool to the external validation cohort, the integrated model classified 56.5% of patients as low risk, maintained a negative predictive value of 83.4%, and successfully identified 74.1% of true OCAD cases.

To our knowledge, this study is the first to apply fully automated plaque quantification on carotid CTA to a large-scale clinical cohort, and to develop and validate a diagnostic model for OCAD based on carotid imaging features. The model can be readily integrated into the existing cerebrovascular assessment workflows to enable concurrent cardiovascular risk screening. However, the study has several limitations. First, the retrospective design and the requirement for paired carotid and coronary CTA examinations within 3 months inherently selected for a higher-risk population. Patients undergoing both examinations likely had greater baseline cardiovascular risk than the general cerebrovascular population. Consequently, the prevalence of OCAD in this cohort may be higher than in unselected patients undergoing isolated carotid CTA, and the model’s generalizability to routine cerebrovascular screening populations requires prospective validation. Second, since both participating centers were from the same national healthcare system and the study population consisted exclusively of Chinese individuals, the findings may not be directly generalizable to non-Chinese populations. Although the model showed consistent performance in a temporally independent validation cohort from the same healthcare system, its applicability to other geographic and ethnic groups requires further validation through prospective, multi-center studies before it can be widely implemented. Third, this study defined the primary outcome (OCAD) as coronary stenosis ≥ 50% on CCTA according to the CAD-RADS criteria. However, CTA provides anatomical information, and anatomically significant stenosis may not necessarily carry hemodynamic significance. Since invasive coronary angiography or functional testing was not used as a uniform reference standard, our model actually predicts anatomically defined stenosis rather than clinically meaningful ischemic disease; therefore, its ability to predict functionally significant ischemia requires further investigation. Fourth, the model’s performance depends on the quantitative accuracy of the automated analysis software employed, and quantitative outputs may vary across different CT scanning equipment, algorithms, and software platforms, which may affect the reproducibility of the proposed workflow when applied to institutions using alternative tools. Therefore, conclusions derived from the software used in this study should be extrapolated cautiously to centers using other platforms, and vendor-specific calibration or algorithm standardization should be considered before widespread implementation. Finally, this study focused on diagnostic performance, and the primary endpoint was the diagnosis of OCAD; we did not assess major adverse cardiovascular events (MACEs). More importantly, we did not evaluate whether the clinical application of this model can improve patient outcomes, reduce unnecessary downstream coronary testing, or provide cost-effective clinical decision support. These issues should be addressed in future prospective studies before this model can be recommended for routine clinical practice.

## Conclusion

In conclusion, this study demonstrates that automated quantitative analysis of plaque components on carotid CTA can effectively identify patients at high risk for OCAD. This model provides a novel, non-invasive adjunctive tool for coronary risk stratification and has the potential to optimize clinical diagnostic pathways. However, it should be noted that the reference standard in this study was anatomical assessment of stenosis on CTA rather than functional assessment of ischemia; thus, the model’s predictive value for hemodynamically significant coronary lesions remains to be further validated. Before routine clinical implementation, prospective multicenter studies are warranted, and its generalizability across different populations and software platforms, as well as its cost-effectiveness, should be evaluated.

## Supplementary information

ELECTRONIC SUPPLEMENTARY MATERIAL

## Data Availability

The datasets generated or analyzed during the study are available from the corresponding author on reasonable request.

## Abbreviations

AUC: Area under the receiver operating characteristic curve
CAD: Coronary artery disease
CTA: Computed tomography angiography
DCA: Decision curve analysis
LASSO: Least absolute shrinkage and selection operator
OCAD: Obstructive coronary artery disease
VIF: Variance inflation factor
