# Supplementary material for: Carotid CTA with automated plaque quantification for risk stratification of coexisting obstructive coronary artery disease
Source: Insights Imaging. 2026 Nov 15;17:295. doi: 10.1186/s13244-026-02472-5 (PMC13769789; doi:10.1186/s13244-026-02472-5)
Supplement: Supplementary file 1 [file 13244_2026_2472_MOESM1_ESM.pdf]

Carotid CTA with Automated Plaque Quantification for Risk Stratification of Coexisting Obstructive Coronary Artery Disease

ELECTRONIC SUPPLEMENTARY MATERIAL

Insights Imaging (2026) Xu L, Zhao M, Zhou C, et al.

Table S1. Comparison of baseline variables between Cohort 1 and Cohort 2

Variables	Cohort 1 (n=2064)	Cohort 2 (n=469)	*p*-value
**Demographics**			
Female sex, n(%)	757 (36.68%)	165 (35.18%)	0.544
Age, years	64.00 (56.00–72.00)	63.00 (55.00–71.00)	0.100
BMI, kg/m²	24.57 (22.43–26.83)	24.41 (22.14–26.73)	0.133
**Cardiovascular risk factors**			
Hypertension, n(%)	1327 (68.72%)	275 (62.64%)	0.014
Diabetes, n(%)	581 (30.43%)	105 (24.08%)	0.009
Smoking history, n(%)	644 (34.38%)	126 (28.97%)	0.031
Cholesterol-lowering drugs, n(%)	1419 (75.44%)	294 (68.85%)	0.005
Dyslipidemia, n(%)	1575 (83.47%)	347 (84.02%)	0.783
**Laboratory indicators**			
Glucose, mmol/L	5.10 (4.60–6.00)	5.30 (4.70–6.40)	0.010
Total cholesterol, mmol/L	4.15 (3.45–4.86)	4.14 (3.40–4.84)	0.797
Triglycerides, mmol/L	1.32 (0.96–1.86)	1.31 (0.95–1.77)	0.642
HDL-C, mmol/L	1.07 (0.90–1.26)	1.00 (0.84–1.17)	< 0.001
LDL-C, mmol/L	2.35 (1.78–2.93)	2.40 (1.81–2.95)	0.755
C-reactive protein, mg/L	1.10 (0.50–3.10)	0.80 (0.50–3.60)	0.177
**Carotid plaque composition**			
Calcified volume	2.64 (1.43-3.50)	2.59 (1.43-3.61)	0.965
Fibrotic volume	1.54 (1.25-2.29)	2.12 (1.31-2.48)	< 0.001
Lipid volume	1.70 (1.21-2.18)	1.21 (1.21-1.63)	< 0.001

Note: Cohort 1 was used for model training and internal validation, while Cohort 2 served as the external validation set. Continuous and categorical variables are presented as described in Table 1 and were compared using the Mann-Whitney U test and Pearson chi-square test, respectively. BMI, body mass index; HDL-C, high-density lipoprotein cholesterol; LDL-C, low-density lipoprotein cholesterol.

Table S2. Collinearity screening for integrated model variables

Variables	VIF
Sex	1.1
Age	1.5
Diabetes	1.1
Cholesterol-lowering drugs	1.2
Calcified volume	2.6
Fibrotic volume	1.7
Lipid volume	1.9

Note: All variables had VIF < 5, indicating no significant collinearity. All variables were retained in the integrated model.

Table S3. DeLong Test for Comparison of Models

Model Comparison	AUC Difference	Z-statistic	P-value
**Training Set**			
Clinical Model vs Plaque Model	-0.063	-4.499	<0.001
Clinical Model vs Integrated Model	-0.078	-7.041	<0.001
Plaque Model vs Integrated Model	-0.015	-3.352	<0.001
**Internal Validation Set**			
Clinical Model vs Plaque Model	-0.072	-3.479	<0.001
Clinical Model vs Integrated Model	-0.088	-5.356	<0.001
Plaque Model vs Integrated Model	-0.016	-2.450	0.014
**Modeling Cohort**			
Clinical Model vs Plaque Model	0.122	9.160	<0.001
Clinical Model vs Integrated Model	0.119	9.018	<0.001
Plaque Model vs Integrated Model	-0.003	-1.434	0.124
**External Validation Cohort**			
Clinical Model vs Plaque Model	0.153	4.238	<0.001
Clinical Model vs Integrated Model	0.145	4.286	<0.001
Plaque Model vs Integrated Model	-0.008	-0.242	0.809

Note: The modeling cohort (from January 2011 to December 2023) was randomly divided into training and internal validation sets at a 7:3 ratio; the external validation cohort was a temporally independent cohort (from January 2024 to June 2025). AUC, area under the curve; Clinical Model: male sex, age, diabetes, and cholesterol-lowering drugs; Plaque Model: calcified volume, fibrotic volume, and lipid volume; Integrated Model: combination of clinical and plaque model variables.

Table S4. Baseline variables of the study population by severe stenosis (≥ 70%) status

Variables	Overall (n = 2064)	Stenosis < 70% (n = 1718)	Stenosis ≥70% (n = 346)	*p*-value
**Demographics**				
Female sex, n(%)	757 (36.68%)	1045 (60.83%)	262 (75.72%)	< 0.001
Age, years	64.00(56.00–72.00)	63.00 (54.00–71.00)	69.00(62.00–75.00)	< 0.001
BMI, kg/m²	24.57(22.43–26.83)	24.54 (22.32–26.90)	24.69(22.55–26.65)	0.683
**Cardiovascular risk factors**				
Hypertension, n(%)	1327 (68.72%)	1058 (66.25%)	269 (80.54%)	< 0.001
Diabetes, n(%)	581 (30.43%)	432 (27.43%)	149 (44.61%)	< 0.001
Smoking history, n(%)	644 (34.38%)	499 (32.66%)	145 (42.03%)	< 0.001
Cholesterol-lowering drugs, n(%)	1419 (75.44%)	1126 (72.55%)	293 (89.06%)	< 0.001
Dyslipidemia, n(%)	1575 (83.47%)	1265 (81.25%)	310 (93.94%)	< 0.001
**Laboratory indicators**				
Glucose, mmol/L	5.10 (4.60–6.00)	5.00 (4.60–5.90)	5.30 (4.80–6.80)	< 0.001
Total cholesterol, mmol/L	4.15 (3.45–4.86)	4.18 (3.47–4.86)	3.95 (3.35–4.90)	0.153
Triglycerides, mmol/L	1.32 (0.96–1.86)	1.32 (0.96–1.87)	1.31 (0.97–1.83)	0.859
HDL-C, mmol/L	1.07 (0.90–1.26)	1.08 (0.92–1.28)	0.98 (0.84–1.17)	< 0.001

LDL-C, mmol/L	2.35 (1.78–2.93)	2.36 (1.79–2.92)	2.29 (1.74–2.96)	0.911
C-reactive protein, mg/L	1.10 (0.50–3.10)	1.00 (0.50–3.00)	1.20 (0.50–3.95)	< 0.001
**Carotid plaque composition**				
Calcified volume	2.59 (1.43–3.61)	2.34 (1.43–3.29)	3.62 (3.06–3.98)	< 0.001
Fibrotic volume	2.12 (1.31–2.48)	1.30 (1.25–2.18)	2.27 (1.63–2.52)	< 0.001
Lipid volume	1.21 (1.21–1.63)	1.52 (1.21–2.10)	2.18 (1.71–2.33)	< 0.001

Note: BMI, body mass index; HDL-C, high-density lipoprotein cholesterol; LDL-C, low-density lipoprotein cholesterol.

Table S5. Univariable and multivariable regression analyses of severe stenosis (≥ 70%)-related variables

Variables	Univariable		Multivariable	
	OR (95% CI)	*P*-value	OR (95% CI)	*P*-value
**Clinical characteristics**				
Female sex	0.50 (0.38–0.65)	< 0.001	0.65 (0.42–0.99)	0.045
Age, years	1.05 (1.04–1.07)	< 0.001	1.00 (0.98–1.02)	0.914
BMI, kg/m²	1.01 (0.97–1.04)	0.683	1.02 (0.97–1.07)	0.540
**Cardiovascular risk factors**				
Hypertension	2.11 (1.58–2.82)	< 0.001	1.09 (0.71–1.67)	0.701
Diabetes	2.13 (1.67–2.72)	< 0.001	1.24 (0.83–1.83)	0.294
Smoking history	1.50 (1.18, 1.90)	0.001	1.05 (0.73–1.51)	0.795
Cholesterol-lowering drugs	3.08 (2.14–4.43)	< 0.001	1.95 (0.92–4.14)	0.083
Dyslipidemia	3.58 (2.24–5.72)	< 0.001	0.97 (0.31–3.06)	0.962
**Laboratory indicators**				
Glucose, mmol/L	1.18 (1.12–1.25)	< 0.001	1.03 (0.94–1.13)	0.492
Total cholesterol, mmol/L	0.91 (0.81–1.03)	0.153	1.19 (0.68–2.09)	0.539
Triglycerides, mmol/L	1.01 (0.90–1.13)	0.859	0.98 (0.76–1.26)	0.872
HDL-C, mmol/L	0.28 (0.17–0.45)	< 0.001	0.24 (0.09–0.60)	0.002
LDL-C, mmol/L	0.99 (0.86–1.15)	0.911	1.00 (0.55–1.81)	0.999
C-reactive protein, mg/L	1.01 (1.00–1.02)	0.001	1.02 (1.01–1.04)	0.001
**Carotid plaque composition**				
Calcified volume	3.29 (2.81–3.85)	< 0.001	2.98 (2.24–3.97)	< 0.001
Fibrotic volume	4.18 (3.32–5.27)	< 0.001	1.08 (0.76–1.55)	0.669
Lipid volume	5.77 (4.34–7.68)	< 0.001	0.89 (0.56–1.42)	0.723

Note: OR, odds ratio; CI, confidence interval; BMI, body mass index; HDL-C, high-density lipoprotein cholesterol; LDL-C, low-density lipoprotein cholesterol.

Table S6. Diagnostic performance of different models for severe stenosis (≥ 70%)

Dataset / Variables	AUC (95% CI)	Sensitivity (95% CI, %)	Specificity (95% CI, %)	Accuracy (95% CI, %)
**Single Variables**				
Male sex	–	24.3 (20.1–29.1)	60.8 (58.5–63.1)	54.7 (52.6–56.8)
Age, years	0.660 (0.631–0.688)	64.7 (59.6–69.6)	59.3 (56.9–61.6)	60.2 (58.1–62.3)
Diabetes	–	44.6 (39.4–50.0)	72.6 (70.3–74.7)	67.7 (65.6–69.7)
Cholesterol-lowering drugs	–	89.1 (85.2–92.0)	27.5 (25.3–29.7)	38.2 (36.1–40.5)
Calcified volume	0.787 (0.758–0.814)	72.5 (67.6–77.0)	71.1 (68.9–73.2)	71.4 (69.4–73.3)
Fibrotic volume	0.720 (0.688–0.757)	80.4 (75.8–84.2)	54.3 (51.9–56.6)	58.6 (56.5–60.7)
Lipid volume	0.731 (0.697–0.765)	60.7 (55.5–65.7)	74.9 (72.8–76.9)	72.5 (70.5–74.4)
**Training Set**				
Clinical Model$_{s}$	0.717 (0.683–0.746)	0.0 (0.0–1.6)	99.7 (99.7–100.0)	83.2 (81.2–85.1)
Plaque Model$_{s}$	0.791 (0.756–0.808)	6.6 (4.1–10.4)	98.6 (97.8–99.1)	83.1 (81.1–85.0)
Integrated Model$_{s}$	0.803 (0.769–0.819)	16.0 (12.0–21.2)	97.8 (96.8–98.5)	84.0 (82.0–85.9)
**Internal Validation Set**				
Clinical Model$_{s}$	0.700 (0.659–0.743)	0.0 (0.0–3.6)	99.3 (99.3–100.0)	83.3 (80.2–86.2)
Plaque Model$_{s}$	0.788 (0.758–0.830)	8.7 (4.7–15.8)	98.8 (97.5–99.5)	83.8 (80.7–86.6)

Integrated Model$_s$	0.790 (0.761–0.831)	10.7 (6.1–18.1)	97.9 (96.2–98.8)	83.3 (80.2–86.2)
**Modeling Cohort**				
Clinical Model$_s$	0.660 (0.631–0.686)	64.7 (59.6–69.6)	59.3 (56.9–61.6)	60.2 (58.1–62.3)
Plaque Model$_s$	0.791 (0.765–0.814)	76.6 (71.9–80.7)	69.9 (67.7–72.1)	71.0 (69.1–73.0)
Integrated Model$_s$	0.790 (0.765–0.814)	74.0 (69.1–78.3)	71.8 (69.6–73.9)	72.1 (70.2–74.1)
**External Validation Cohort**				
Clinical Model$_s$	0.623 (0.567–0.686)	87.8 (79.4–93.0)	33.3 (28.7–38.1)	43.7 (39.3–48.2)
Plaque Model$_s$	0.801 (0.755–0.846)	64.4 (54.2–73.6)	82.9 (78.7–86.3)	79.3 (75.4–82.8)
Integrated Model$_s$	0.802 (0.753–0.847)	65.6 (55.3-–74.6)	82.1 (77.9–85.6)	78.9 (75.0–82.4)

Note: The modeling cohort (from January 2011 to December 2023) was randomly divided into training and internal validation sets at a 7:3 ratio; the external validation cohort was a temporally independent cohort (from January 2024 to June 2025). AUC, area under the curve; The subscript "s" indicates the model established in the sensitivity analysis. Clinical Model$_s$: male sex, age, diabetes, and cholesterol-lowering drugs; Plaque Model$_s$: calcified volume, fibrotic volume, and lipid volume; Integrated Model$_s$: combination of clinical and plaque model$_s$ variables. AUC was not calculated for single binary variables (e.g., sex, diabetes).

Supplemental Figures:

Figure S1. Variable selection was performed using LASSO logistic regression

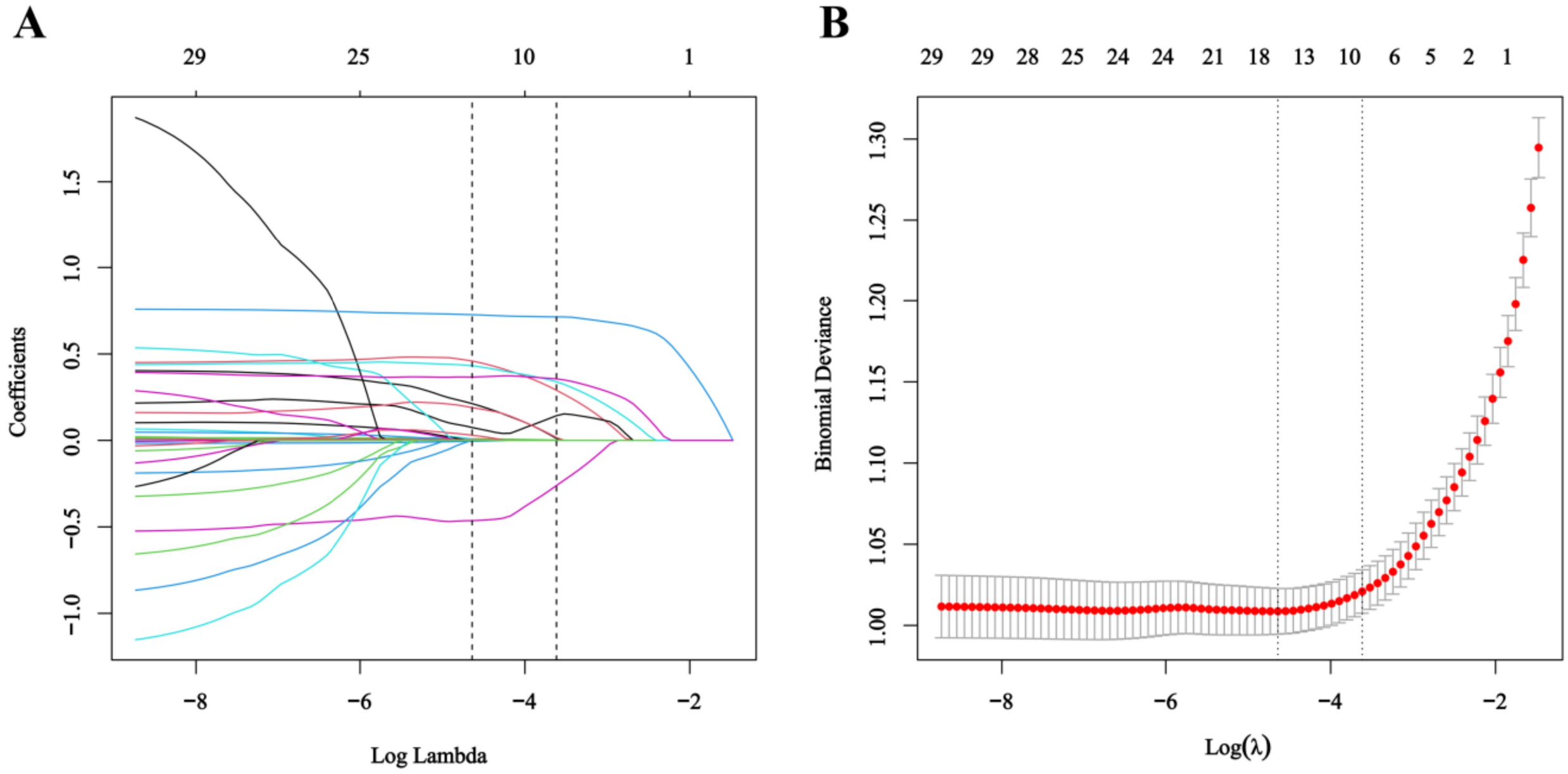

Note: A The tuning parameter (λ) in the LASSO model was determined via 10-fold cross-validation based on the one-standard-error criterion. **B** Coefficient profile plot showing the paths of coefficients against λ.

Figure S2. ROC Curves Comparing Three Models Across Different Datasets

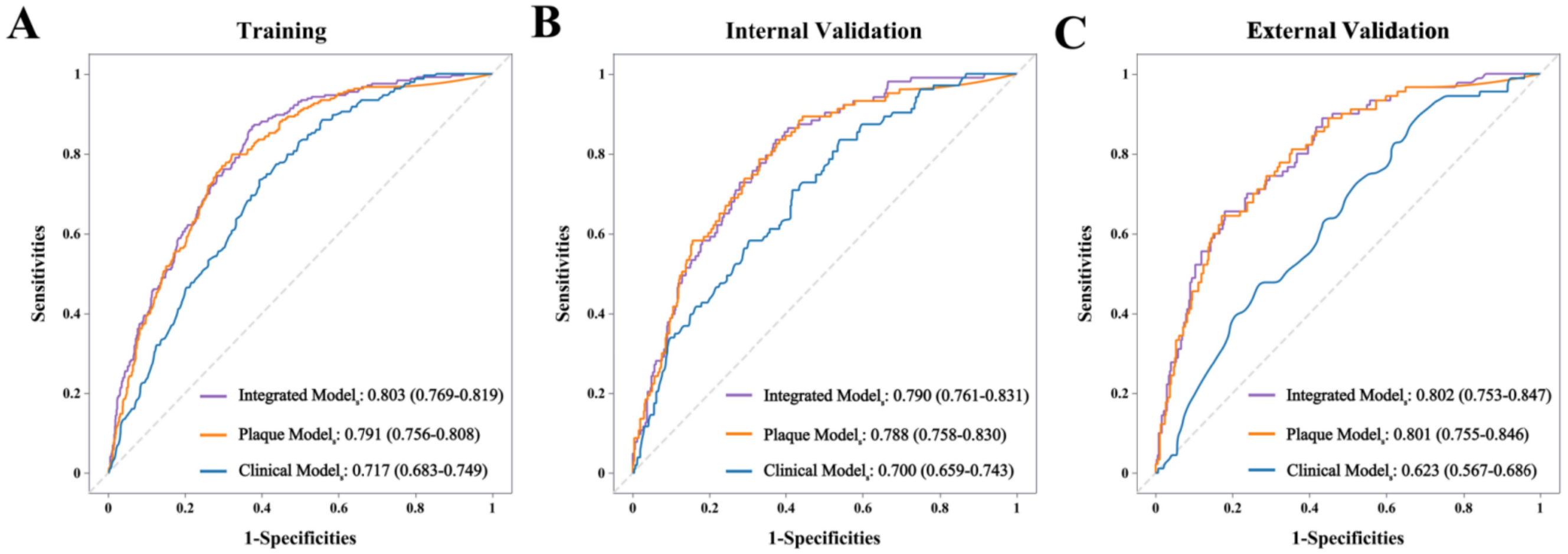

Note: The ROC curves of the three models in the sensitivity analysis on the training set (**A**), internal validation set (**B**), and external validation set (**C**). AUC, area under the curve; The subscript "s" indicates the model established in the sensitivity analysis. Clinical $Model_s$: male sex, age, diabetes, and cholesterol-lowering drugs; Plaque $Model_s$: calcified volume, fibrotic volume, and lipid volume; Integrated $Model_s$: combination of clinical and plaque $model_s$ variables.

Figure S3. Decision Curve Analysis and Calibration Curves Comparing Three Models Across Different Datasets (Sensitivity Analysis)

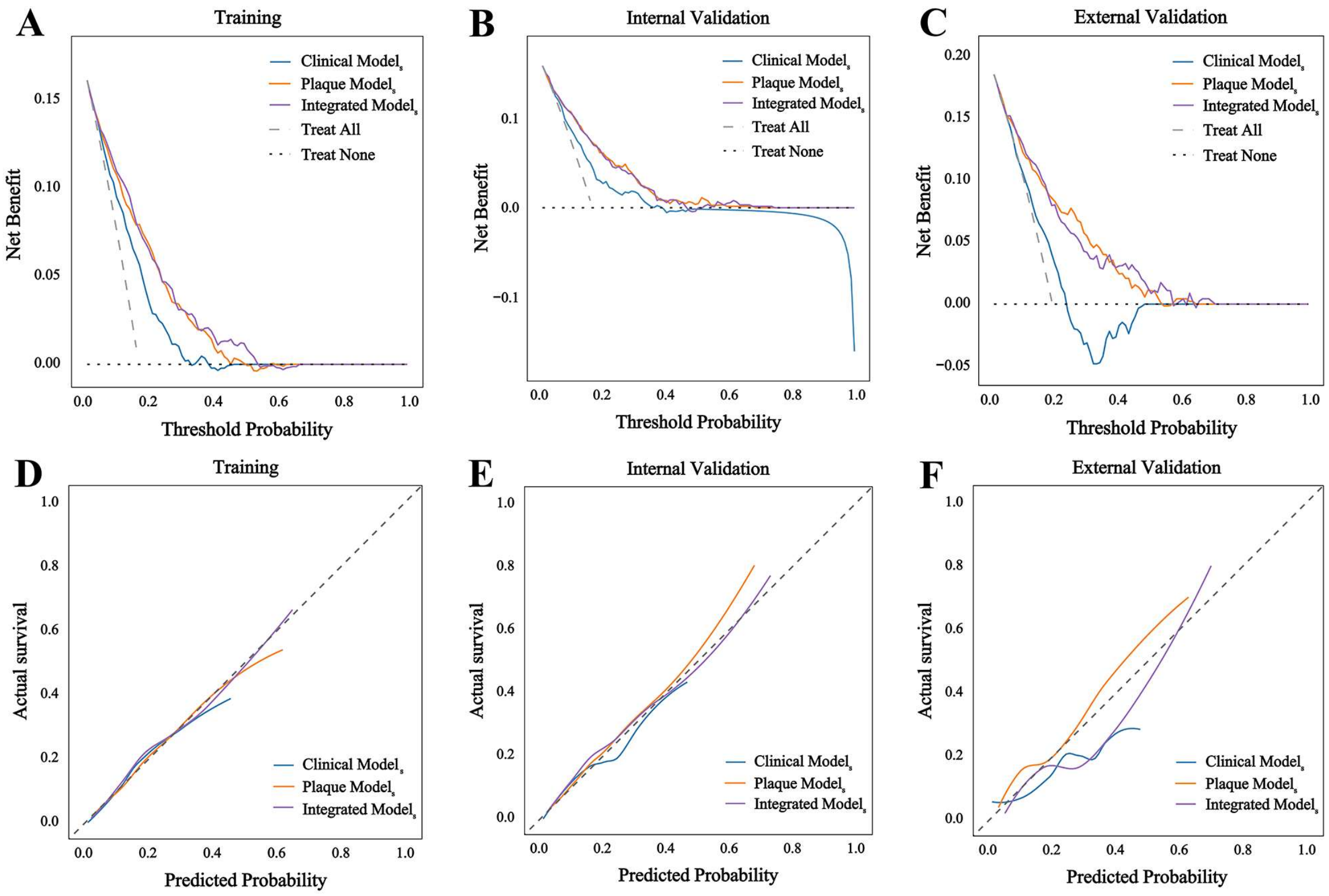

Note: The subscript "s" indicates the model established in the sensitivity analysis. **A–C** Decision curve analysis of the three models in the training set (**A**), internal validation set (**B**) and external validation set (**C**), respectively. **D–F** Calibration curves of the three models in the training set (**D**), internal validation set (**E**) and external validation set (**F**), respectively.
